# Supplementary material for: A mathematical modelling framework for the regulation of intra-cellular OCT4 in human pluripotent stem cells
Source: PLoS One. 2021 Aug 4;16(8):e0254991. doi: 10.1371/journal.pone.0254991 (PMC8336844; doi:10.1371/journal.pone.0254991)
Supplement: S1 File — (PDF) [file pone.0254991.s001.pdf]

# S1 File.

**Supporting information file for the manuscript: A mathematical modelling framework for the regulation of intra-cellular OCT4 in human pluripotent stem cells**

L E Wadkin, S Orozco-Fuentes, I Neganova, M Lako, N G Parker, A Shukurov.

**S1 Appendix** **The Hurst Exponent.** The Hurst exponent, $0 < H < 1$, is a measure the self-similarity properties of a time series [1, 2]. Here we use the MATLAB® function *genhurst* [3] which estimates $H$ using re-scaling methods [4, 5]. Further details on the Hurst exponent, other methods of estimation and its relation to fractional Brownian motion can be found in Refs. [1, 2, 6, 7]. We calculate the Hurst exponent for every cell with an OCT4 time series of length 50 or more (1119 cell cycles). The distribution of $H$ is shown in S1(a) Fig with the standard deviation error each $H$ calculation in S1(b) Fig. The log-log re-scaled range plot (from which $H$ can be estimated as the gradient) with the mean $H = 0.38$ fitting line and a 95% confidence interval is shown in S1(c) Fig.

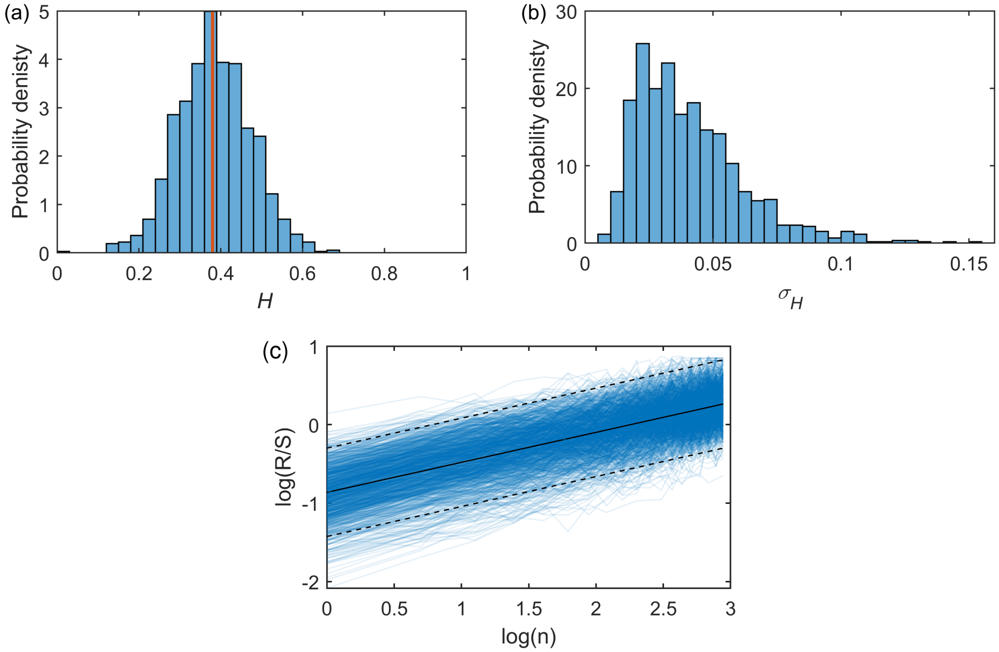


**S1 Fig. Experimental Hurst exponents.** (a) The distribution of all Hurst exponents, $H$, for all cells in the experiment with an OCT4 time series of 50 points or more (1119 cell cycles). The orange line shows the mean of $H = 0.38$. (b) The standard deviation, $\sigma_H$, of each Hurst exponent calculation. (c) The log-log rescaled range ($R/S$) with time series index ($n$) for all 1119 cells considered. The mean Hurst exponent $H = 0.38$ fitting is shown as a black solid line, with dashed lines showing the 95% confidence interval for the fitting.

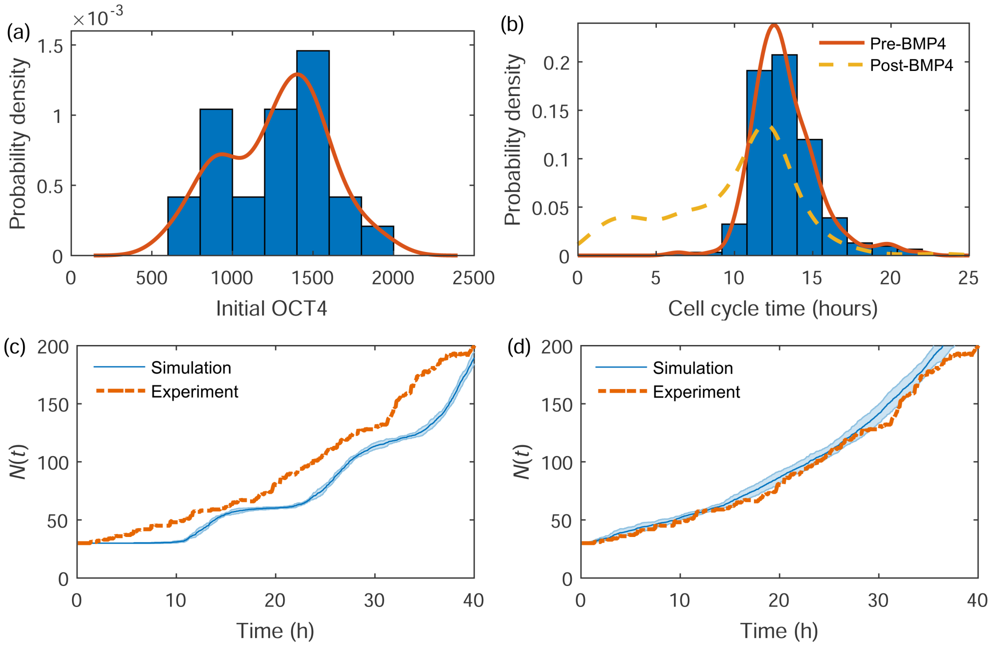


**S2 Fig. The initial conditions and resulting population dynamics for the common base model.** (a) The distribution of experimental initial OCT4 values histogram, OCT4$(t = 0)$, with kernel density fitting shown in orange. (b) The experimental distribution of cell cycle duration times histogram for all cells pre-BMP4 addition with kernel density fitting (orange). The corresponding fitting for all cells post-BMP4 is shown in yellow-dashed. The number of cells over time, $N(t)$, when cellular division is (c) synchronised and (d) not synchronised in step 3 of the common base model. Blue solid lines show the simulated population sizes with standard deviation error range shown in light-blue (calculated over five realisations) and orange dashed lines show the experimental population.

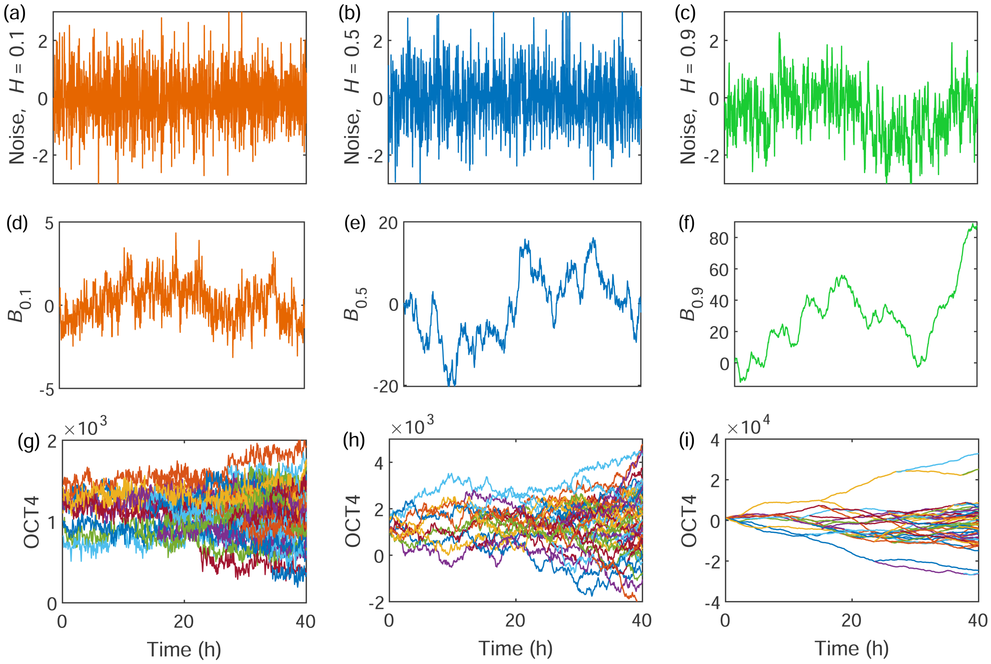


**S3 Fig. The effect of the Hurst exponent.** Realisations of simulated noise in fractional Brownian motion with (a) $H = 0.1$ (anti-persistence), (b) $H = 0.5$ (Brownian) and (c) $H = 0.9$ (persistence), (d-f) the corresponding simulated trajectories with initial condition $B_H$ $(0) = 0$. (g-i) Simulation of OCT4 for 40 hours, with ten initial cells, and temporal OCT4 determined by simulated realisations of $\sigma B_H$ with $\sigma = 90$ and (g) $H = 0.1$, (h) $H = 0.5$ and (i) $H = 0.9$.

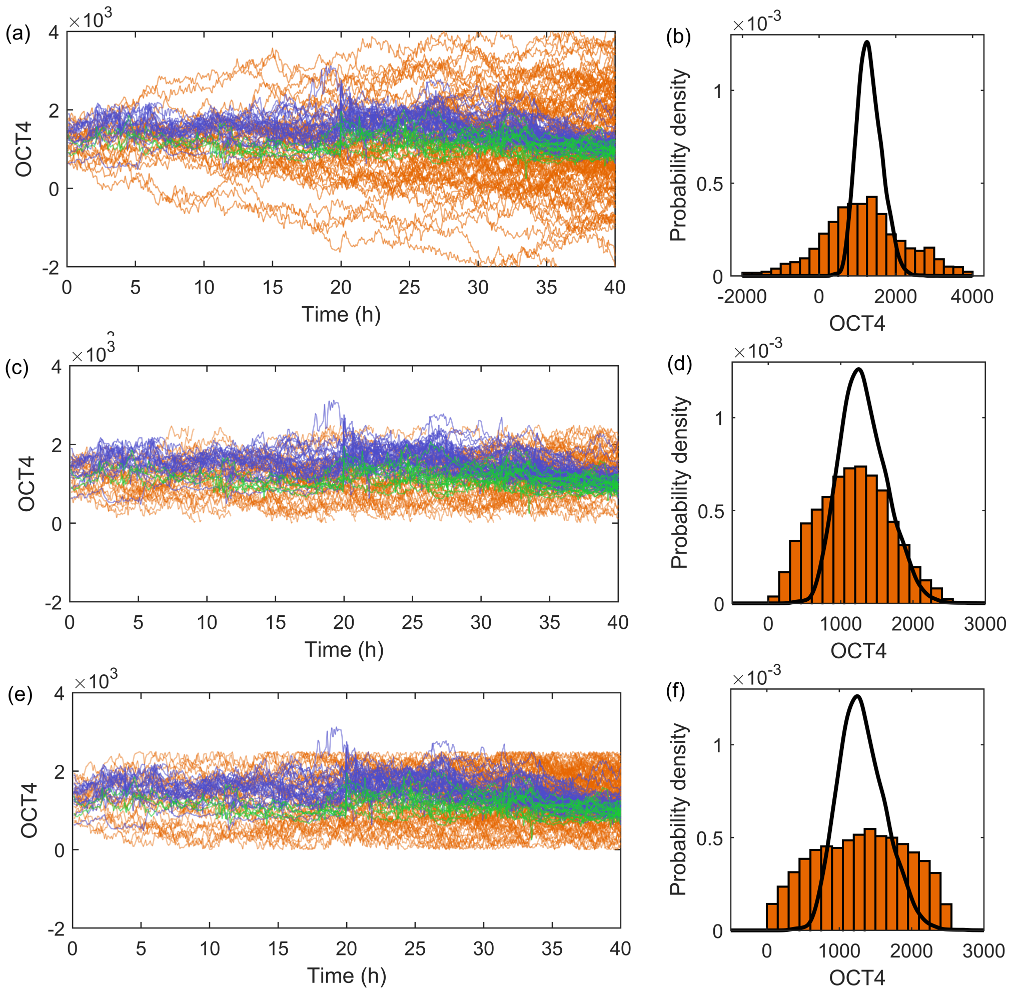


**S4 Fig. The effect of boundary conditions on simulated OCT4.** Simulated OCT4 expression (orange) using fBm with 16 initial cells, $\sigma = 90$ and $H = 0.38$ with (a) no, (c) absorbing, and (e) reflecting boundary conditions at zero and 2500 (to correspond to the boundaries which contain 99% of the experimental data). The experimental data is overlaid in purple (pluripotent cells) and green (differentiated cells). The corresponding histograms for simulated OCT4 (orange) with (b) no, (d) absorbing, and (f) reflecting boundaries. The kernel density fitting to the experimental distribution is shown in black.

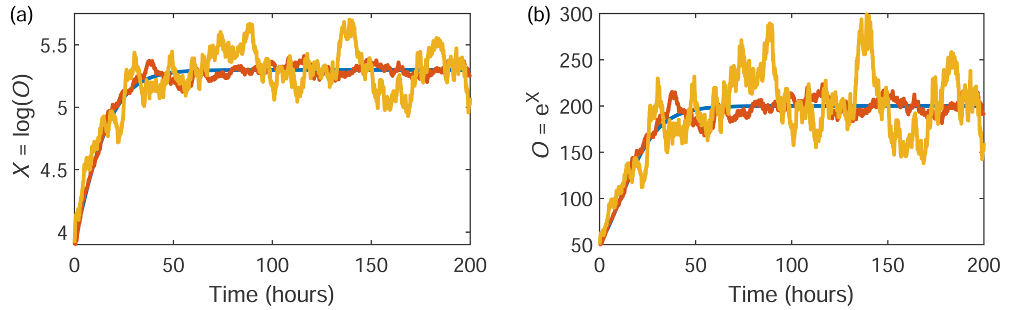


**S5 Fig. The SLE with multiplicative noise.** Realisations of the dynamics of (a) $X = \log(O)$ and (b) $O = e^X$ from Eq (3) with $r = 0.1$/h, $K = 100$, $\xi = W_t$ and $\sigma_{\mathrm{M}} = 0$ (blue), 0.025 (orange) and 0.075 (yellow).

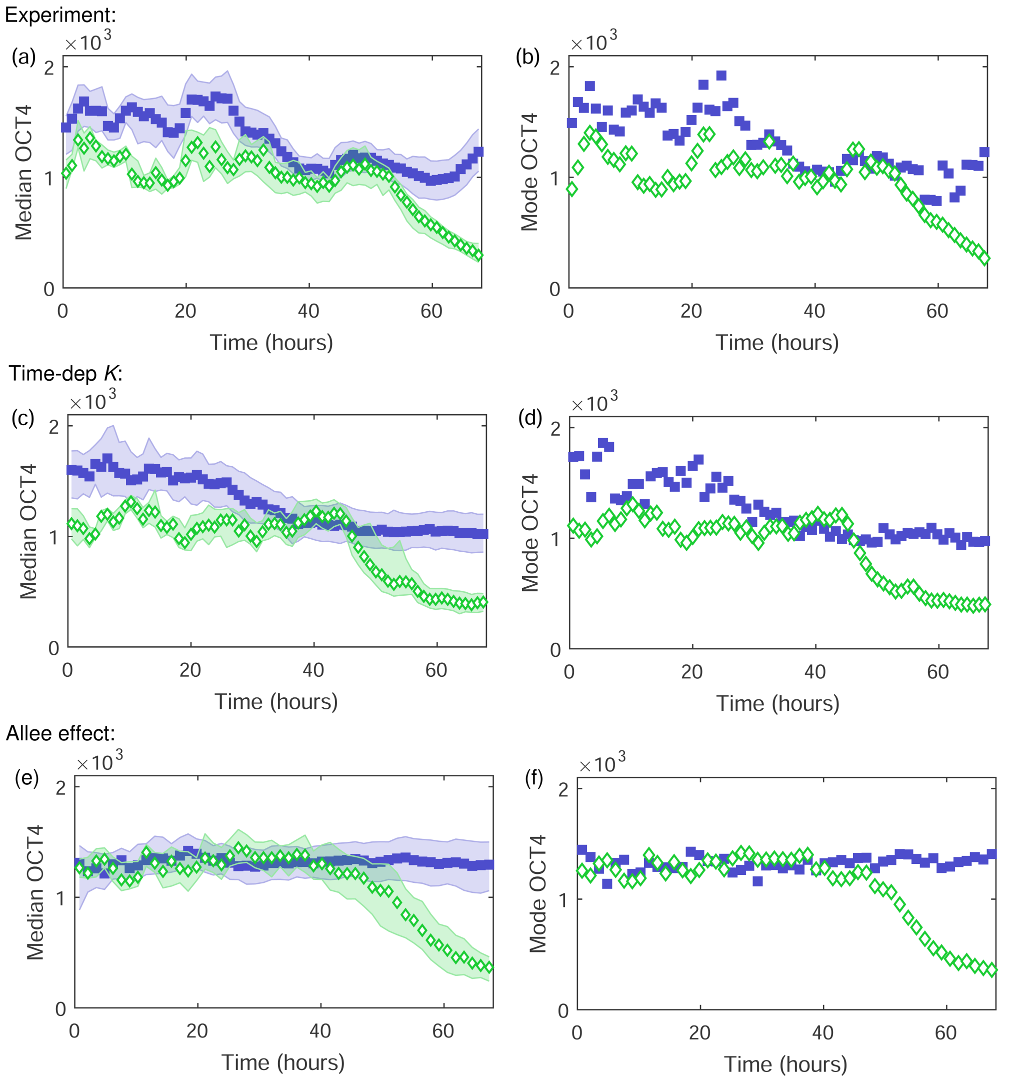


**S6 Fig. The median and mode experimental and simulated OCT4 expressions using a time-dependent carrying capacity and an Allee effect.** The (a,b) experimental and (c-f) simulated median and mode OCT4. In (c,d) the dynamics are simulated using the SLE with additive and multiplicative noise, and a time-dependent carrying capacity, Eq (5), with parameters specified in Table 3. In (e,f) the dynamics are simulated using the SLE with an Allee effect at 43 hours, Eq (7), with $r = 0.025$, $K = 1290$, $\sigma_{\mathrm{A}} = 35$, $\sigma_{\mathrm{M}} = 0.035$ and $A = 1000$.

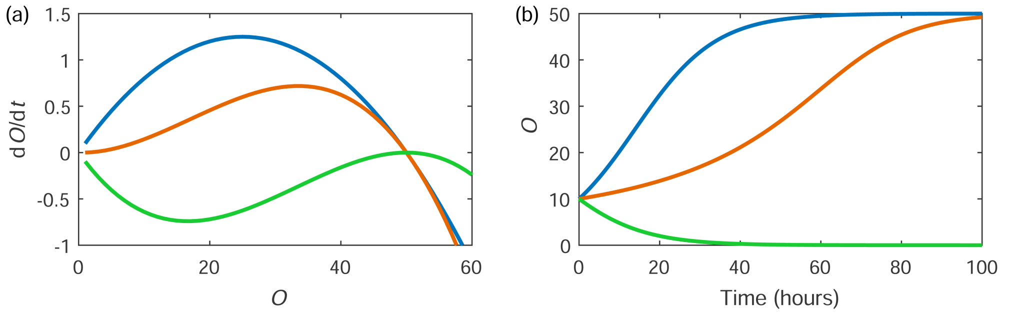


**S7 Fig. The deterministic logistic equation with a demographic Allee effect.** The deterministic logistic equation with an initial condition of $O_0 = 10$, $r = 0.1\,/\text{h}$, $K = 50$ and an Allee effect, Eq (6), for (a) $dO/dt$ and (b) $O$ with $A = 1$ (orange) and $A = 50$ (green). The deterministic logistic equation with no Allee effect is shown in blue.

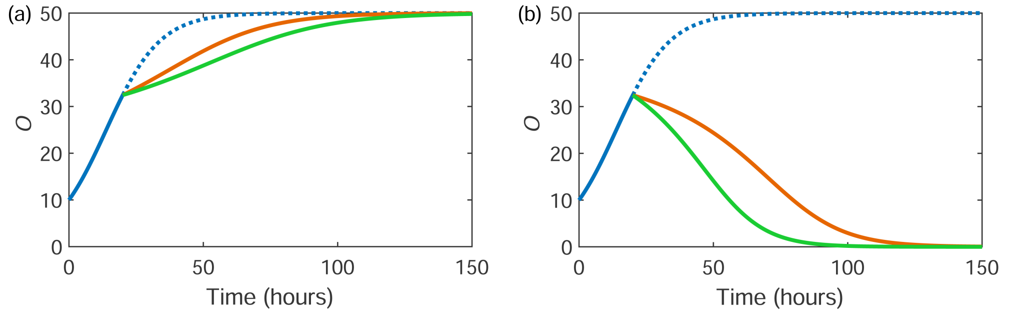


**S8 Fig. Switching on a demographic Allee effect.** The deterministic logistic equation with an initial condition of $O_0 = 10$, $r = 0.1\,/\text{h}$ and $K = 50$ (blue). The Allee effect term in Eq (6) is introduced at $t = 25\,\text{h}$ with (a) $A = 20$ (orange) and $A = 25$ (green) and (b) $A = 40$ (orange) and $A = 50$ (green). The deterministic logistic growth with no Allee effect is shown as blue dashed.

## References


1. Mandelbrot BB, Van Ness JW. Fractional Brownian motions, fractional noises and applications. SIAM Rev. 1968;10(4):422–437.

2. Mielniczuk J, Wojdyłło P. Estimation of Hurst exponent revisited. Comput Stat Data Anal. 2007;51(9):4510–4525.

3. Aste T. Matlab File Exchange: Generalized Hurst exponent; Accessed: 21.09.2019. Available from: `https://uk.mathworks.com/matlabcentral/fileexchange/30076-generalized-hurst-exponent`.

4. Matteo TD. Multi-scaling in finance. Quant Finance. 2007;7(1):21–36. doi:10.1080/14697680600969727.

5. Matteo TD, Aste T, Dacorogna MM. Long-term memories of developed and emerging markets: Using the scaling analysis to characterize their stage of development. J Bank Finance. 2005;29(4):827–851. doi:https://doi.org/10.1016/j.jbankfin.2004.08.004.

6. Lacasa L, Luque B, Luque J, Nuno JC. The visibility graph: A new method for estimating the Hurst exponent of fractional Brownian motion. EPL. 2009;86(3):30001.

7. Barunik J, Kristoufek L. On Hurst exponent estimation under heavy-tailed distributions. PHYSICA A. 2010;389(18):3844–3855.
