# Supplementary material for: A mathematical modelling framework for the regulation of intra-cellular OCT4 in human pluripotent stem cells
Source: PLoS One. 2021 Aug 4;16(8):e0254991. doi: 10.1371/journal.pone.0254991 (PMC8336844; doi:10.1371/journal.pone.0254991)
Supplement: Attachment — Submitted filename: Response to reviewers.pdf [file pone.0254991.s002.pdf]

Response to reviewers

We are grateful to the referees for their insightful and constructive comments on our manuscript (*A mathematical modelling framework for the regulation of intra-cellular OCT4 in human pluripotent stem cells* PONE-D-21-04839. Below we address each of their comments in turn and list the major changes in the revised manuscript.

Reviewer 1

Wadkin et al. take an already published 68h long experimental dataset of the OCT4 expression (from Wolff et al., 2018) and aim to mathematically model the exact OCT4 expression in each cell of this lineage tree. Note that last year, Wadkin et al. published a related theoretical paper on the same dataset. The authors start with an intuitively simple base model consisting of N=16 cells with OCT4 level and cell cycle duration taken from the corresponding experimental distributions. They test a plethora of model variations with the aim to capture the exact experimental data. In the end, the degree of complexity and the number of parameters go beyond reasonable. In general, this manuscript reads more like a personal learning experience than a research article. This study does not have a high impact, neither is it original. I would recommend rejecting it, as a minimum major revision is needed.

Major points:

1. Line 110 and lines 166-174: Since the entire manuscript builds on the finding H=0.38, please show the derivation of the Hurst exponent, i.e., fit on a log-log plot, in the supplement. In the discussion, please compare this value to other studies.

   Since we obtained a Hurst exponent for every time series in the dataset (1274 cell cycle) we have not given details of the fits of each individual Hurst exponent. However, we agree that some further context on this calculation would be valuable. We now more clearly state that this was an average calculation of H for every time series (in the main text lines 117-120 and Supplementary Information), provide the formal definition of the Hurst exponent (in SI), and the distributions of H for all cells (Figure S1). In addition to this, we have also provided the standard deviations from each calculation of the Hurst exponent to indicate the accuracy of each of these estimations (Figure S1). The error in the average of all H is also now stated (line 113-114). We include comparison to other studies calculating the Hurst exponent for gene expression in bacteria in the discussion (lines 494-499). We are not aware of any other studies that calculate H in stem cell gene expression.

2. Fig 2c: Why is it at all needed to report a synchronized model? That being said, it looks like Fig 2c matches better if all cells have undergone exactly half their cell cycle at the start of the model. Shifting the blue curve six hours to the left makes a synchronized and non-synchronized look qualitatively similar, making it harder to say that the non-synchronized is the better of the two.

   Thank you for raising this important point. Since we do not know the initial cell cycle stages of the cells, they could reasonably lie somewhere between the two options shown in Figure 2 (now S2) (b) and (c). We agree this is not of primary importance in this context so have moved this Figure to the Supplementary Information. To show our decision making process in building the model we have added further clarification on this point (lines 168-175).

3. Line 191-211: Both absorbing and reflecting boundary conditions introduce two new parameters that are not mentioned nor discussed. The lower boundary is reasonably set at 0. However, the higher boundary is ill-defined, and the results in Fig S2c-f depend on this parameter. In general, the arguments for introducing absorbing and reflecting boundary conditions are weak.

   We chose to begin with the exploration of boundary conditions as they are a standard feature of stochastic models and the simplest possible choice to achieve some form of regulation as a first step. We agree that the imposition of these boundary conditions raises further biological questions (lines 248-252) however the exact placement of the boundaries does not impact the choice of statistical modelling in a significant way. It is for the lack of biological certainty that in the next sections we then seek to introduce a different method of regulation to capture the range of OCT4 seen in the experiment. To make this process clearer, we have further stressed the problem with boundary conditions (lines 248-252) and in the discussion (lines 512-515). The estimation of the top upper boundary, in particular, as you say, would require further examination. In this case it is approximated as 2500, with 99.9% of the data points falling beneath this value experimentally (we now also state this in the corresponding Supplementary Figure S4 and the main text, lines 230-233). Since the OCT4 is measured in arbitrary fluorescence units, we might also reasonably expect this value to change with different experimental and imaging conditions. We believe it is still worth discussing the introduction of boundary conditions to tell the story as to why we have not used them as the primary method of controlling the stochastic fluctuations.

4. Line 248: Please, describe what is r? What does it represent? Please elaborate on how this parameter is estimated. Specifically, in line 248, r=0.02 is used, vs. in line 265, r=0.005 is used.

   We have added some further discussion regarding the parameter r (lines 283-288), describing its function as a growth rate parameter in the logistic equation. In the stochastic version, it controls how tightly the realisations are regulated back towards the carrying capacity - off-setting some of the effect of the stochastic noise term σ. It is for this reason r is different between the simulations including different types and levels of noise. In our simulations we have r as a free parameter and it has been chosen to achieve qualitatively similar results to the experimental data (added in lines 283-288). Future work could further constrain this parameter (along with the parameter sigma) using more formalised parameter search methods. This links with our further discussion added regarding the number and constraints of parameters in response to points 7 and 8.

5. After line 279: What is additive and multiplicative noise in this biological system? Specify and exemplify. Why is it important to consider both types of noise in this case? Please elaborate on why additive noise might be dominant at early times and multiplicative at late times. What occurs to the cells when they transition from additive to multiplicative noise?

   Thanks for raising this important point. We now further clarify additive and multiplicative noise in relation to genetic expression upon their introduction (lines 271-274 and 301-303) and in the discussion (lines 532-537). Our choice of testing out a noise transition was lead by the experimental data showing the temporal skew which became apparent at around 20 hours. To link each type of noise specifically to other intra-cellular reactions would require further work to identify which types of noise are dominant at different times (and the biological reactions these correspond to). In our model we are coarse graining these intra-cellular processes, so the next step would be to explore the individual processes taking place. This could be linked

to experimental results which show that when OCT4 production is high (at early stages in the cell cycle, Ref. 17) the system does not take into account the current levels of OCT4 in the cell (new Ref. 69). We have now highlighted this would be an interesting avenue of future work (lines 341-343 and in the discussion, lines 544-548 and 557-561).

6. Line 283: The time point 20h seems completely arbitrarily chosen. The same applies to the 25h in line 303. What scientific biological arguments underlie these time points?

   The time point 20h is chosen as the time of the noise switch as the time when the distribution of OCT4 becomes positively skewed in the experimental data, estimated from calculating the skew of the distributions in Figure 1c. This time point is an adjustable parameter which can be chosen to reflect the experimental data considered. It is true that this chosen time point is somewhat arbitrary as we would not expect a sudden change, but rather a smooth transition, and this could be further refined in future studies. This is now clarified in the text, lines 350-353. The time point 25h is chosen as the time to initiate a reduction on OCT4 based on the average OCT4 values over time plots (Figure S6). The carrying capacities for each are then calculated from the data pre and post this time point. We have clarified this choice in the text, lines 372-374. In both of these cases, further investigation could result in more sophisticated choices of these 'switches', with instead functions for the noise and changing K (although this will add more parameters to set these functions, line 385-388 and 586-588). These time points could also be linked with other experimental results which show changes in OCT4 at different stages through the cell cycle (Ref 17).

7. Table 1: The number of parameters has now grown beyond 10. Overall, the authors need to use solid scientific arguments for evaluating whether a model captures the experiments (rather than saying it is qualitatively similar, line 269) or is better than another model (rather than saying it is an improvement, line 272). Occams razor and how to fit an elephant become relevant. This comment applies to the rest of the manuscript.

   Linked to comment 8, we have added further clarity on which parameters are free and which are constrained within the tables. We feel it is not appropriate at this stage to quantitatively compare the two choices for differentiation models, since they require different starting assumptions (based on whether we want to capture a stochasticity in which cells become differentiated, or whether we would rather specify these prior). The models are descriptive and each capture different aspects of the data. We agree that in future work and for predictive models, these more formal comparisons would be useful. We now highlight in the discussion (lines 601-606) that in future model development formal methods (such as Bayesian Information Criteria) would be useful in evaluating and comparing the two models further.

8. Line 323: While this is part of the experimental dataset and it is a motivation for this study, it takes up very little space in the manuscript, and it blows up the number of parameters to approximately 20. Please streamline the story and minimize the number of parameters. Please distinguish between those parameters that are free vs. those that are constrained, and present a parameter scan of the free parameters.

   Thank you for this useful suggestion. We believe the extension of the models discussed in the earlier sections to include cell differentiation is the next intuitive step. Naturally, since it is a direct extension of previous models it requires less space to introduce. We agree that the number of parameters may appear large since we are considering two populations with different behaviours, particularly for the shifting carrying capacity case which requires

changes in K with time. However, many are constrained by the data (i.e., N_0, H, K). We now streamline the presentation of the parameters, including only the fitting parameters in the tables and have added further clarity on which parameters are free and which are constrained. We have added discussion on this issue on lines 617-619, clarifying that although the number of parameters increases the flexibility of the model (e.g., by being able to capture both the pre-BMP4 decrease in the pluripotent cells and the post-BMP4 decrease in the differentiated cells) it is a downside to a shifting carrying capacity model, and future work would focus on putting further constraints on the currently free noise and growth rate parameters. We feel a parameter scan of the free parameters in the models is outside the scope of this work, however suggest it as a future step in model development, along with inference of model parameters from experimental data (lines 617-619).

9. Line 526: Can it be true that only one study reports OCT4 expression in hESC? To the best of my knowledge, there are other published datasets available that at least deserve to be mentioned, especially If the authors attempt to develop a general mathematical model.

   We have added reference to several studies which report OCT4 expression in hESCs, Refs [70-72]. However, the dataset used is still the most appropriate starting point due to its tracking of multiple cell cycle generations at very short discrete time intervals. We would be very keen to know of more experimental data of this type if it has missed our searches.

10. The discussion reads more like an extended summary than an actual scientific discussion.

    We hope our additions to the discussion from both reviewers comments have improved this.

11. Please explain why a Gillespie model is never applied?

    We could have applied a Gillespie model to simulate stochastic trajectories from the differential equations involved, breaking down the logistic equation into a series of reactions, however, this would have introduced another parameter (splitting up r into two parameters). There are also advantages to avoiding deriving the stochastic DE from Markov jump processes, as they provide less flexibility over the non-demographic noise within the system (doi:10.1016/j.biosystems.2014.05.002). We have added new references to the discussion (Refs. 65-67) on lines 544-548 for the Gillespie model and note that it could be useful when linking the data to chemical reactions but gives less freedom over the noise, with new Ref. 68.

12. Please, make the MATLAB scripts generating the models and the figures fully available.

    These have been uploaded to 10.25405/data.ncl.14474412 (currently under review with the repository but should be available shortly).

Minor points:

1. Abstract line 3: known is misspelled.

   Corrected.

2. The abstract is lacking a conclusion.

   Agreed, corrected.

3. Fig 1a: Why is this figure not the same as Fig 2a in Ref18?

   We plot raw data from their available dataset. Note that Ref 18 uses a log scale and includes the unknown cells.

4. Fig 1b-d: What do the authors want to show by including these panels? Please comment on these in the main text or remove them from the manuscript if they are not strictly needed to tell the story.

   These panels outline the key temporal features we view to be important to capture through the modelling process. These are commented on in the main text in the bullet pointed list (beginning line 112). We later examine these distributions from the resulting models and believe it is helpful to give an initial overview to familiarise readers with the key features of the data.

5. Fig 2a-b: It is easier to compare the orange curve and the blue bars by plotting CDFs and not PDFs.

   Agreed, we now show the CDFs in the main text. We have kept the PDFs in the Supplementary Information (new Figure S2, along with Fig 2c-d, relating to the cell cycle synchronisation).

6. Fig 2b: Does this distribution change with time or cell state?

   Good question - we choose the cell cycle distribution based on all cells before BMP4 treatment to set up our initial conditions. There is no significant difference (confirmed by the Kolmogorov-Smirnov test at the 95% level) between the pre-BMP4 pluripotent and differentiated cells. However, there is a statistically significant different between the pre- and post-BMP4 cell cycles, with the post-BMP4 cells showing a greater proportion of cells with a shorter (between 0–10 hours) cell cycle. For simplicity and because colony growth is not the main feature of the manuscript, we choose to keep the same distribution throughout. We have added a note on this in the text in lines 172-174 and added the post-bmp4 distribution Figure S2(b).

7. 30 cells in line 79 contra 16 cells in line 188? Please clarify the distinction.

   Here we are not considering the cells in the 'unknown' category. The colony starts with 14 pluripotent, 2 differentiated and 14 unknown cells. This has been clarified in the text, line 96.

8. Line 83: Position information is not essential for this study. There is no need to state it.

   Agreed, removed.

9. Line 87: It might be more meaningful to state the mean and standard deviation cell cycle duration.

   Agreed, now stated on line 87.

10. Line 92: What defines the unknown category? Please be more specific about how the pluripotent and differentiated states are defined. If specific thresholds are used, then mention them.

    We have added further details to the text on lines 89-94 under Experiment summary).

11. Line 121: What is the number of cells in each colored-curve in Fig 1c? Please provide those numbers and a statistical test on the statement in this line number.

    The number of cells is now included in the Figure. We also make reference to Supplementary Figure S6 to illustrate the change in mode throughout time. We use the Kolmogorov-Smirnov test to show the difference between each distribution is significant (now stated on lines 128-129.)

12. Line 134: Here, please restate the purpose of developing these models.

    Included (now line 141).

13. Line 152: What is the required number of division events for OCT4 cells? Please state it.

    Thanks, we agree this could be made clearer. The OCT4 series are generated for every cell across its whole lifetime (not at each timestep) and so input is the number of division events (or the number of cells we want to include) rather than timesteps. This has been clarified in the text on lines 163-165.

14. Line 180: Why is the built-in MATLAB function not used over the homemade function?

    The built-in function is available through the Wavelet toolbox, which we did not have access to.

15. Lines 191 and 194: This should be Fig S2a specifically. Also, be more specific in lines 205 and 211.

    Corrected throughout.

16. Line 206-210: These sentences are hard to read. Please, rephrase.

    Corrected.

17. Line 450-452: Why are chemical reactions additive noise? Please, explain.

    Reworded and added further reference to the text on lines 532-537. At the initial stages of the cell cycle when the TF regulatory network is active we expect the noise does not depend on the current state of the system (Ref. 69).

18. Ref42 is a webpage. When was it last updated, and when was it accessed?

    Thanks, corrected.

Reviewer 2

Oct4 is a key regulator of pluripotency. In this work, the authors explore stochastic models that can describe experimental data on Oct4. The study is based upon a previous publication where Oct4 dynamics in a colony of human embryonic stem cells were followed by time-lapse fluorescence microscopy, prior to and after the addition of the differentiation-inducing signal BMP4. Based upon the expression of the marker Cdx2, cells are classified as either pro-pluripotent or pro-differentiated over the whole lineage. A range of increasingly complex phenomenological stochastic models are presented, and their ability to describe key features of the data is examined. All the models are based upon simulating oct4 dynamics over a cell cycle, independently of cell cycle length, which is chosen from the experimental distribution of cell cycle durations. Ultimately, the authors show that a model based upon a logistic equation with a time-dependent Allee effect, additive and multiplicative noise, can reproduce almost all the key features of the data.

The paper is very clear, the mathematical assumptions well explained and the conclusions are critically presented.

Minor points:

1. Although the logistic equation is found to be an effective phenomenological equation to reproduce key features of the data, it deviates from the common reasoning about pluripotency factors as regulating each other with feedback interactions. I would encourage the authors to discuss a bit more what other model forms could be used to account for some of the biological knowledge known, and whether maybe this would simplify the noise terms. In particular, I wonder if the antipersistence modelled by the fBm noise could also emerge as a result of negative feedback, where increases in oct4 are followed by decreases, and vice-versa, and whether in this case simpler noise models may be enough. If there is any prior work pointing to this it would be interesting to highlight.

   Thank you for the thoughtful comment, we agree. We mention that the carrying capacity in the logistic equation could represent some of these feedback interactions with other pluripotency factors (rather than being entirely separate from these methods, it side-steps the need to define all of the regulatory pathways). We have extended this to discuss other stochastic models which use network feedback interactions instead and more clearly emphasised how the role of the carrying capacity could encapsulate these relationships in lines 619-627. With comparable experimental data for other regulation genes it would be possible to develop these links within the model. We see both types of models being valuable, each with their advantages.

2. In ref. 33, a prior publication on the same data by the same authors, and in the original experimental publication, the authors report that BMP4 is added at t=43h, whereas in the present work it says treatment is added at t=40h (line 80, line 99). This should be fixed or the discrepancy clarified.

   Thank you, agreed. Corrected to 43 hours.

3. I was not able to find the data/code in the data.ncl so I suppose this will be done prior to publication.

   The data is available through the original publication, and our code is now available at 10.25405/data.ncl.1447441 (currently under review with the repository but should be available shortly).

Major changes:

- Inclusion of new Figure S1 and Appendix S1 detailing the calculation of the Hurst exponent.
- Stastistical tests given to confirm temporal distributions are significantly different.
- Figure 2 now shows the cdf plots with Figure S2 showing the pdfs and the cell synchronisation.
- Discussion added on cell cycle synchronisation.
- Refined boundary conditions section and discussion of their biological implications.
- Parameter tables now show only fitting parameters and indicate which of these are free and which are constrained by the experimental data.
- Further clarification added on the types of noise and biological implications.
- Additions to discussion including: Hurst exponents for other genetic expressions, ways to simulate stochastic series, next steps in parameter inference and the links with other network models with feedback loops.
- New references: Ref. 46, 55, 56, 65-73.
