# Supplementary material for: A mathematical modelling framework for the regulation of intra-cellular OCT4 in human pluripotent stem cells
Source: PLoS One. 2021 Aug 4;16(8):e0254991. doi: 10.1371/journal.pone.0254991 (PMC8336844; doi:10.1371/journal.pone.0254991)
Supplement: Attachment — Submitted filename: Response to reviewers #2.pdf [file pone.0254991.s003.pdf]

Response to reviewers

We are again grateful to the referees for their insightful and constructive comments on our manuscript (*A mathematical modelling framework for the regulation of intra-cellular OCT4 in human pluripotent stem cells* PONE-D-21-04839). Below we address each of their comments in turn.

Reviewer 1

Last time I argued for rejecting this manuscript because too many w1changes would be needed to make it publishable. I did not find the manuscript of high impact nor - and more critical to PLOS One - original. Since then, the authors have improved the manuscript. However, major changes are still required. In particular, to make this study publishable in PLOS One, it must be valid. I write comments related to the validity as major points below with note that these are not time-consuming but critical for publication in PLOS One. Other remarks of less importance to PLOS One are provided as minor points.

Major points:

1. Abstract lines 4-5: 'drug discovery' is not a clinical application, and 'personalized medicine' is overshooting the perspectives of hPSCs and the long-term perspectives of the manuscript. Sentences like these oversell the manuscript. Please down tune. The abstracts of references 1-6 do not even mention personalized medicine.

   Agreed, our motivation in mentioning these studies was to set the background for why hPSCs are important in general, rather than relating to our results in particular - especially since the work is inter-disciplinary and mathematicians may not be aware of the applications of hPSCs. We have streamlined the abstract, introduction, and the aforementioned references accordingly.

2. I cannot run the scripts to reproduce Figs 1-2 due to an unrecognized field name, 'Fate'. Please provide the cell data struct directly in the repository with all required field names. Also, I cannot reproduce Fig 6 among others due to an unknown function or variable 'shadedErrorBar'. Please fix all these errors.

   We originally chose not to upload the data file as it belongs to another group. However, since this study (Ref. 14) is open access, we have now uploaded to cell data struct with a note of its origin for ease of being able to reproduce our figures. The error bar function is also now included.

3. Unsatisfactory, the authors still do not present a log-log H curve. Given what the authors present, I am unsure whether the authors exclude any cells with few time points from the H estimation. The shortest time series of a cell in the dataset is 0.25h = 3 time points. Do the authors also calculate an H value for them? The genhurst command recommends at least 50-time points for reliable H estimation.

   We now show the log-log H curve in the Supplementary Information. We present log-log H lines for all cells included on one axes against the mean fitting of H with a 95% fitting confidence interval. Thank you for your note about the time series length. Indeed, the Hurst exponent is only calculated for cells with 50 time points (1119 cell cycles). We now state this in the manuscript when introducing H and in the Supplementary Information.

4. Lines 90-93: Please show this in the supplement or refer to it if it's already published.

   Thanks, we now refer to the data set publication for this.

5. Line 488: I would not call these 'statistical models' conceptual or descriptive maybe, but not statistical. No statistical tests have been applied, and the models have not been used in any quantitative way.

   We have changed this to refer to 'stochastic models'.

6. Lines 548: I cannot see how ref 68 backs up this statement. Please elaborate or remove the statement along with the reference.

   We have decided to remove as this is a statistical tangent from the rest of our discussion. We were referring in general to Markov jump processes such as those arising from birth death processes with the reference showing that SDEs derived from MJPs did not adequately describe their data due to a reduction in flexibility around noise control.

7. Figs 1a and 3a: Please keep colors consistent across (all) figures. Is it correct that the orange lines in Fig 3a are identical to the blue lines in Fig 1a? And if so, why is there an orange line above 2500 at 39h in Fig 3a and no blue line at the same intensity and time in Fig 1a?

   Originally we used purple for pluripotent cells, and green for differentiated cells throughout the manuscript, unless we are considering the cell fates together, in which case we use orange. For example in Figure 3a since our model does not consider pluripotent and differentiated cells separately, we chose to present both experimental cell fates in orange (with simulated ones in blue). However, we agree this switch in colours could cause confusion. Now we have edited this to stick to the original purple and green introduced (with colours now changed in Figure 3 and S4), with an extra note regarding the model considering both cell fate types together. Thank you for spotting the discrepancy in Fig 3a, this data had been plotted including the 'unknown fates' and has now been fixed.

8. Fig 5 caption: 'For pro-pluripotent cells, the carrying capacity reduces at 20 hours, whilst the carrying capacity for pro-differentiated cells is constant.' If I am right, this does not match the information provided in Table 2. In Table 2, K is reduced at 25h for both cell types!

   Apologies for this mistake. We have changed the Figure caption accordingly.

9. Figs S7-S8 and Eq. 6: I am getting confused in the notation. What is N? Relative to O? What is the value for N in Fig S7? And what is the value for O in Fig S8? Both refer to Eq. 6 that contains N and O, but N is never introduced in the main text.

   Many thanks for this. This is a mistake left over from our original introduction of the demographic Allee effect using population size (N). We have since changed to describe it in context using the variable O for OCT4. We have updated the caption and axes labels to reflect this.

10. Tables 2-3: For differentiated cells, K=1000 after 25h (and $<$ 43h) in Table 2, whereas in Table 3, K=1100 after 25h (and $<$ 43h) for differentiated cells as well. Is this difference in the K value made on purpose? And why? No comment is provided on this in the main text.

    This slight reduction in carrying capacity in differentiated cells was originally not used in this model to simplify the number of changes in the carrying capacity overall (the change between 1100 and 1000 is minor compared to the reduction upon differentiation to 300), but we agree

this is not clear. We have now edited this to keep it consistent with the previous section, keeping $K = 1000$ for differentiated cells post-25 hours before the reduction for differentiation.

11. Tables 2-3: Changing sigma A and sigma M from Table 2 to Table 3 makes it difficult to compare Fig 5 with Fig 6. What is the argument that these parameters that affect the simulations < 43h should change when introducing an experimental change at 43h? Please comment on this change in the main text or be consistent with the choice of parameter values.

    Agreed, we now keep the values of sigma the same between Table 2 and Table 3 and have updated all figures after these parameter adjustments (it does not affect the results).

Minor points:

1. Abstract: What do the authors mean by 'important aspects' and 'distinct advantages'? Please specify. Also, the use of 'sophisticated' seems odd.

   Edited.

2. Line 86: "Denote the times between(?) cell birth and division"

   Rephrased.

3. Line 88: Please remove additional "."

   Thanks, but we cannot spot this typo on this line.

4. Line 128: Please list the supplementary figures in the order they are mentioned. Fig S6 is mentioned before Fig S2.

   Corrected.

5. Line 132: change "shown" to "show" and line 133: change "shown" to "as shown."

   Thanks, corrected.

6. Line 163: To increase reproducibility, the authors could consider stating the exact number of division events used as requested already during the last round of peer-review, i.e., 200 for diff cells and 500 for pp cells, etc.

   Apologies for missing this point before. We now state on when discussing the base model the number of events required to comfortably cover the whole experimental time frame for both groups. For shorter time frames, the number of events can be reduced, or the model can be run to this excess number of events with the extra time-frames not of interest removed at the end.

7. Lines 297-298: Please elaborate on the statement in these two lines.

   Edited.

8. Line 302: What do the authors mean by "other cellular components"?

   We have removed the word 'other' here - could be any cellular component.

9. Lines 512-514: This is also imprecise and vague. Please either eliminate or elaborate on what "variety of experimental and imaging conditions" the authors mean.

   We have removed this statement and a similar one in the Results section.

10. Lines 601-606: These lines are based on my previous comment. However, I am not satisfied with the authors' reply. Although this is not strictly needed to make the manuscript publishable in PLOS One, I would appreciate, if the authors early on in the manuscript introduced a few success criteria that the models need to satisfy to make them 'good' models. The authors need a reliable way to compare the models, whether this is a Bayesian, statistical, or descriptive comparison. In Table 4, the authors give it a try. However, these 'features' are not presented early on in the manuscript nor clearly in Table 4.

    As you say, we are aiming to provide a descriptive comparison of the features, initially presenting the key features that the models should capture (in 'Temporal OCT4 features') and then exploring models which are capable of reproducing these features descriptively. We now re-emphasise this point when introducing these temporal features, now labelling each feature F1-6 and mapping the models to each feature when we present them in the Results section. We have also edited Table 4 to present this more clearly.

11. Fig 1a-b: Please add legends to (all) figures to increase readability, especially for Fig 1a-b.

    This is now included.

12. Fig 3b caption: Please write "estimated carrying capacity (vertical line, K = 1290)"

    Edited.

13. Figs S6 and S9: Why are the authors presenting the mode? What information does the mode add that the median does not? Often the authors refer only to panel S6c but not S6a; why? Actually, in line 373, the mean and median are mentioned in parenthesis, but S6 shows the mode and the median.

    We chose to include the mode for completeness since some of the OCT4 distributions through time are skewed, and so the mean, median and mode can all be different. We now present the information of Figure S6 and S9 together in Figure S6 only and have tidied up our references to Figure S6, including the typo in line 373.

Reviewer 2

All the comments have been addressed. Just be aware of a couple of typos, in line 513 and line 515-516.

Thank you, the typos have now been corrected.
